# A Prospective, Interventional, Comparative Study to Evaluate the Efficacy of Using Combined Platelet-Rich Plasma and Platelet-Rich Fibrin Over Standard Cleaning and Dressing in Chronic Wounds

**DOI:** 10.7759/cureus.70092

**Published:** 2024-09-24

**Authors:** Sparsh Gupta, Anand Zingade, Mayur Baviskar, Shrikant V Pingale

**Affiliations:** 1 General Surgery, Yashwantrao Chavan Memorial Hospital, Pune, IND; 2 General Surgery, Pimpri Chinchwad Municipal Corporation's Postgraduate Institute, Yashwantrao Chavan Memorial Hospital, Pune, IND; 3 Plastic and Reconstructive Surgery, Pimpri Chinchwad Municipal Corporation's Postgraduate Institute, Yashwantrao Chavan Memorial Hospital, Pune, IND

**Keywords:** chronic non-healing wounds, conventional dressing, platelet-rich fibrin (prf), platelet-rich plasma (prp), platelet-rich plasma (prp) and platelet-rich fibrin (prf) chronic wound management, therapeutic dressing, therapeutic interventions

## Abstract

Introduction: Chronic wounds are defined as wounds that have failed to proceed through the orderly process that produces satisfactory anatomic and functional integrity or that have proceeded through the repair process without producing an adequate anatomic and functional result. The majority of wounds that have not healed in three months are considered chronic, although a duration as low as four weeks has been used to indicate chronicity. Our study aimed to compare the efficacy of autologous platelet-rich plasma (PRP) and platelet-rich fibrin (PRF) versus standard cleaning and dressing as a regenerative medicine strategy to promote healing in chronic wounds.

Methods: A prospective randomized controlled trial was undertaken to test the efficacy of autologous PRP and PRF in the healing of chronic wounds. A series of 60 cases was compiled from patients attending the outpatient department regularly for the management of chronic wounds. A total of 30 cases were randomly chosen for study with autologous PRP and PRF and 30 cases received conventional dressing.

Results: The average healing duration in the study was significantly shorter for the PRP & PRF group. The mean healing time for this group was 4.45 weeks (31.2 ± 3.07 days) compared to 9.61 weeks (67.27 ± 9.19 days) for the conventional dressing group.

Conclusion: PRP and PRF belong to a new generation of platelet concentrates that help efficaciously for enhanced healing and functional recovery, safely and cost-effectively. They help by shortening the recovery period overall, improving the quality of life of patients, and altogether eliminating the additional morbidity of operative procedures.

## Introduction

Chronic wounds are defined as wounds that have failed to proceed through the orderly process that produces satisfactory anatomic and functional integrity or that have proceeded through the repair process without producing an adequate anatomic and functional result. The majority of wounds that have not healed in three months are considered chronic, although a duration as low as four weeks has been used to indicate chronicity [1].

Wound healing is a complex process of overlapping phases that is initiated by an injury or wound. Normal wound healing is divided into phases defined by characteristic cellular populations and biochemical activities: (a) hemostasis and inflammation; (b) proliferation; and (c) maturation and remodeling. All wounds need to progress through this series of cellular and biochemical events to successfully re-establish tissue integrity. However, multiple factors can interfere with this sequence and can lead to prolonged healing (chronic wounds) or non-healing [1].

The management of chronic ulcers presents a formidable challenge, owing to the recurrent trauma, compromised vascularity, and/or excessive inflammation that perpetuates the chronicity of the wound. The primary objective of chronic wound treatment is to promote wound healing, prevent complications such as amputation, and achieve optimal outcomes. Conventional management strategies encompass local debridement, dressing changes, vacuum-assisted closure (VAC), and surgical interventions. Furthermore, eliminating infection through appropriate antibiotic coverage, optimizing nutritional support, and managing co-morbidities are essential adjuncts to wound care. Despite these comprehensive efforts, a significant number of chronic wounds remain refractory to healing.

Newer treatment modalities include stem cells, platelet-derived growth factors (PDGFs), fibrin glues, etc., which increase the response in healing chronic wounds. The use of blood derivatives was suggested in the last 50 years for the treatment of chronic skin wounds. Fibrin matrix and platelet components (particularly growth factors) offer interesting healing properties as surgical adjuvants and can be obtained quickly and at low cost.

Platelet-rich plasma (PRP) is a bioactive product characterized by a concentrated platelet content, obtained through centrifugation of autologous whole blood. Upon activation, PRP releases a plethora of factors that play a crucial role in tissue repair and regeneration [2]. Due to its straightforward preparation, abundant growth factor content, and minimal immunogenic potential, PRP has been extensively used in various therapeutic applications and is currently being investigated for its efficacy in treating chronic wounds [3-5].

Platelet-rich fibrin (PRF) constitutes a pioneering biomaterial that leverages the therapeutic properties of blood constituents, yielding a singular matrix that integrates platelet and immune cell concentrates within a unified fibrin membrane [6]. Distinct from conventional fibrin sealants and platelet concentrates, PRF presents an autologous, self-sustaining entity that emulates the physiological wound healing matrix, generated through unadulterated centrifugation of blood without extrinsic additives or manipulation [7].

Due to a lack of sufficient literature, our study aimed to compare the efficacy of autologous PRF and PRP versus standard cleaning and dressing as a regenerative medicine strategy to promote healing in chronic wounds.

This article was previously presented as a paper at the 2023 ASICON, 83rd Annual Conference of The Association of Surgeons of India, in Visakhapatnam, Andhra Pradesh, India on 13th December 2023.

Objectives

The primary objectives of the study were to assess the effectiveness of PRP and PRF compared to conventional cleaning and dressing methods in enhancing early wound healing in chronic wounds. The evaluation focused on two key outcomes: healing rate, measured by indicators such as dressing soakage, granulation tissue color, wound edge status, and surrounding skin condition; and healing duration, defined as the time required for the wound to become suitable for grafting or achieve re-epithelialization.

## Materials and methods

The study was conducted in the Department of Surgery of Pimpri Chinchwad Municipal Corporation’s Postgraduate Institute, Yashwantrao Chavan Memorial Hospital, Pimpri, Pune from June 2023 to December 2023 by a single surgeon under the guidance of a senior surgeon. A prospective randomized controlled trial was undertaken to test the efficacy of combined usage of autologous PRP and PRF in the healing of chronic wounds. A series of 60 cases were compiled from patients attending the outpatient department regularly for the management of chronic wounds. A total of 30 cases were randomized using research randomizer software for study with autologous PRP and PRF and 30 cases received conventional dressing. The study was conducted with the ethical approval of the institutional review board, ensuring adherence to the ethical standards in research involving human subjects.

The following formula was used for the sample size calculation: n = (Zα/2+Zβ)2*2*σ2/d2. Where, Zα/2 is the critical value of the normal distribution at α/2 (e.g., for a confidence level of 95%, α is 0.05 and the critical value is 1.96), Zβ is the critical value of the normal distribution at β (e.g., for a power of 80%, β is 0.2 and the critical value is 0.84), σ2 is the population variance = 11.36 = 3.372, and d is the difference you would like to detect = 2.58 weeks [8,9]. The minimum required sample size will be 29 per group.

Selection criteria

The study's inclusion criteria comprised patients aged 18 years and above presenting with chronic wounds, who had undergone dressing for a duration exceeding three months, and who exhibited treated varicose veins and controlled diabetes mellitus. Conversely, the exclusion criteria consisted of pregnant females, individuals undergoing anti-platelet therapy, seropositive patients, and those with laboratory results indicating hemoglobin levels below 10 mg/dl, serum albumin levels below 2.5 gm/dl, or platelet counts below 100,000 platelets per microliter.

Preparation of material

Preparation of PRP

The preparation of PRP involves a three-step process. Initially, blood is collected from an individual through venipuncture into a tube containing an anticoagulant, such as acid citrate dextrose and sodium citrate solution. This is followed by centrifugation at 3000 revolutions per minute (rpm) for 15 minutes, resulting in the separation of the blood sample into three distinct layers: the bottom layer comprising red blood cells and leukocytes, the middle layer containing PRP, and the top layer consisting of platelet-poor plasma (PPP). A double centrifugation process is typically employed, with the first spin (hard spin) separating PRP from red blood cells and the second spin (soft spin) distinguishing PRP from PPP. To activate PRP, inducers of aggregation such as bovine thrombin and 10% calcium chloride are utilized, stimulating degranulation and the release of growth factors. This activation process increases platelet concentration by three to five times within 15 minutes compared to native plasma.

Preparation of PRF

**Figure 1:**
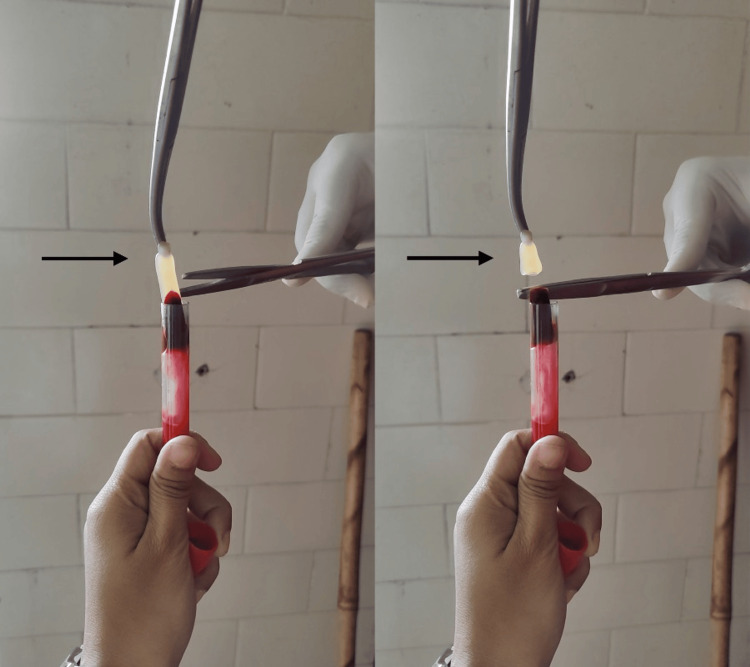
Preparation of platelet-rich fibrin (black arrow).

The protocol for preparing PRF is straightforward and requires the same equipment as PRP. Approximately 5 ml of whole venous blood is collected in two sterile 6 ml vacutainer tubes without anticoagulant. Following centrifugation at 3000 rpm for 10 minutes, the blood separates into three distinct layers: a lower red fraction containing red blood cells, an upper straw-colored cellular plasma, and a middle fraction comprising a fibrin clot. The upper layer is removed, and the middle fraction is collected, specifically the 2 mm section below the dividing line, which constitutes the PRF. This process involves the concentration of fibrinogen in the upper part of the tube, which combines with circulating thrombin during centrifugation to form fibrin, resulting in a fibrin clot in the middle of the tube. This clot entraps platelets within its fibrin meshwork (Figure 1).

Dressing technique

A total of 60 patients were enrolled in the study, and each participant provided informed consent subsequent to receiving a comprehensive explanation of the procedure in their native language. Prior to randomization, all 60 wounds underwent debridement. Following debridement, the wounds were randomly allocated to either the experimental group (case group) or the control group, receiving standard care.

For PRP and PRF Group

**Figure 2:**
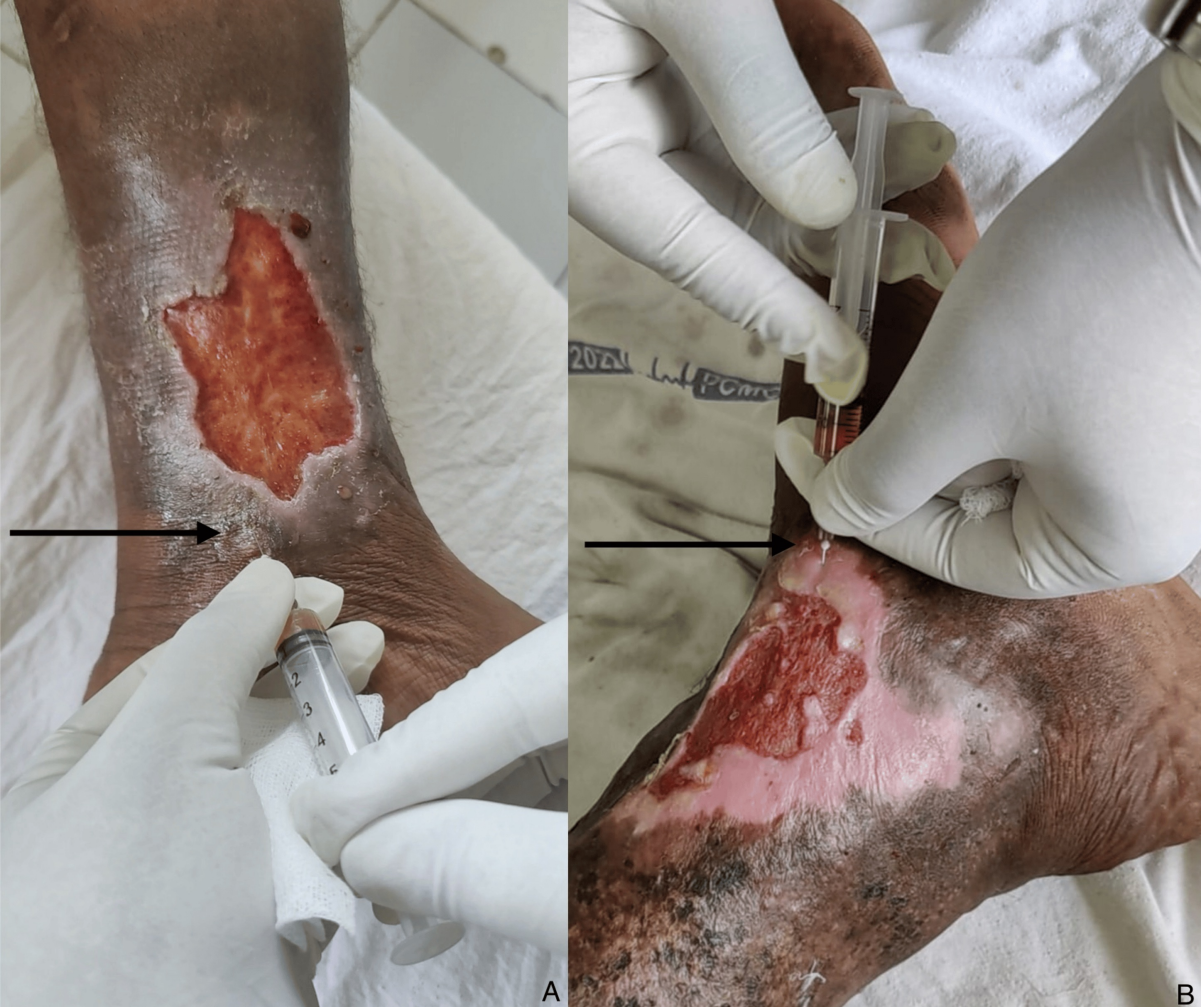
Injecting platelet-rich plasma (PRP) into wound edges. The image shows injecting PRP (black arrow) in two different wounds (A & B).

**Figure 3:**
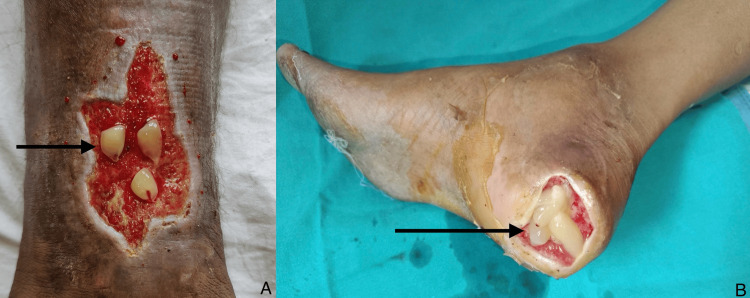
Application of platelet-rich fibrin (PRF) over ulcer. The images show PRF application over ulcers in two wounds (A & B).

**Figure 4:**
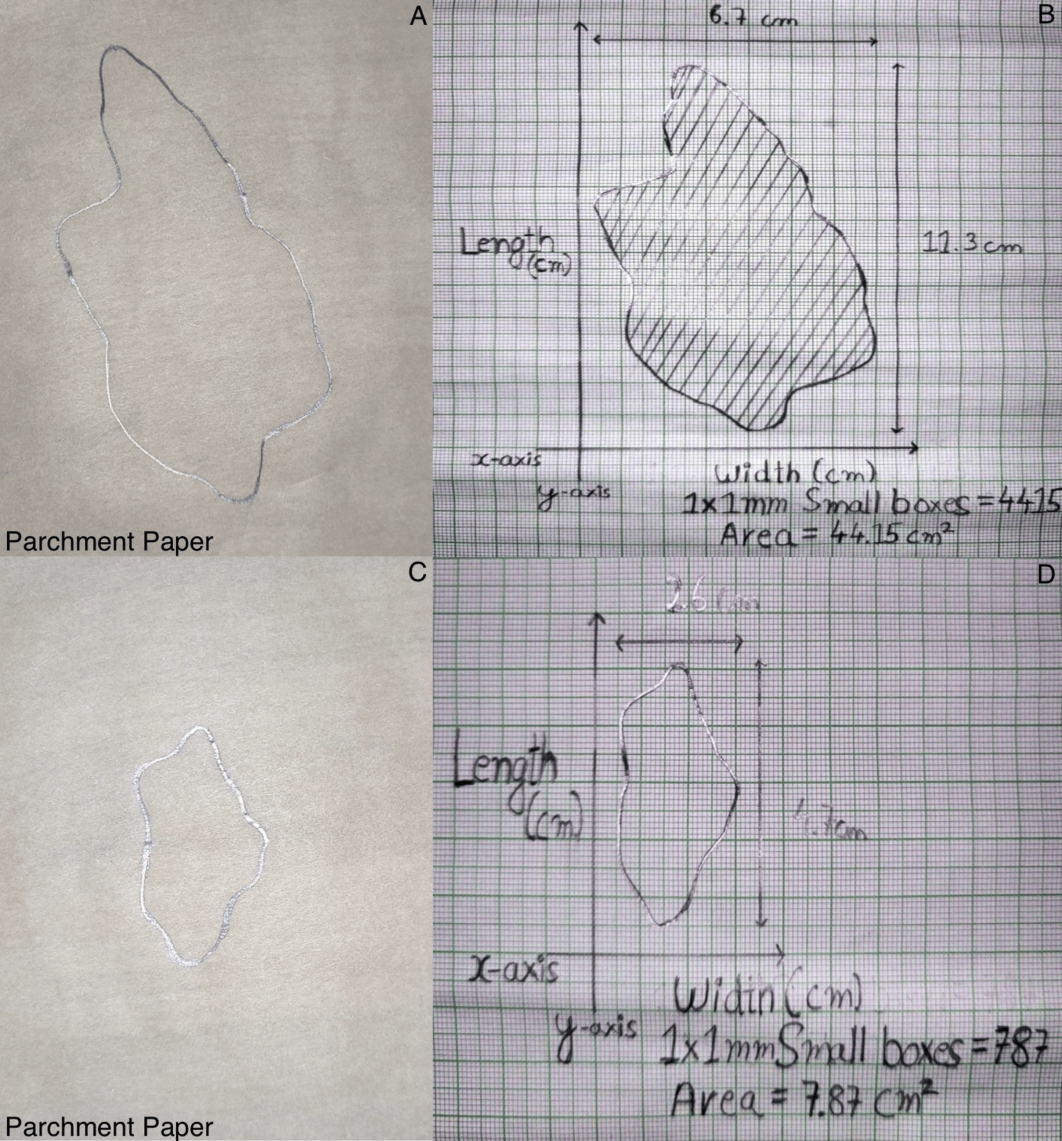
Calculation of wound area. The images show (A) parchment paper imprint of the wound on day one, (B) area calculation by plotting on graph paper, (C) parchment paper imprint of the wound on day 21, and (D) area calculation by plotting on graph paper.

The patient's ulcer was meticulously cleaned with sterile normal saline solution. Subsequently, autologous PRP and PRF were prepared from the patient's blood. The PRP was administered via injection into the wound edges using a 26-gauge needle (Figure 2), followed by the application of PRF over the ulcer site (Figure 3). The wound was then covered with a chlorhexidine-impregnated sheet and sterile gauze dressing. Dressings were changed at three-day intervals, during which time wound parameters were documented. Ulcer size was quantified by creating a parchment paper imprint, which was then graphed on a bar chart to calculate the wound area in square centimeters (Figure 4).

For the Conventional Group

Patients in the control group received sterile dressing with a chlorhexidine-impregnated sheet and saline-soaked gauze after cleaning with normal saline.

## Results

The study aimed to evaluate the effectiveness of the combined usage of PRP and PRF over conventional cleaning and dressing with normal saline. The comprehensive data collected from 60 patients offer significant insights into the patient demographics, clinical outcomes, and the benefits of using PRP and PRF over conventional treatments.

Patient demographics

**Figure 5:**
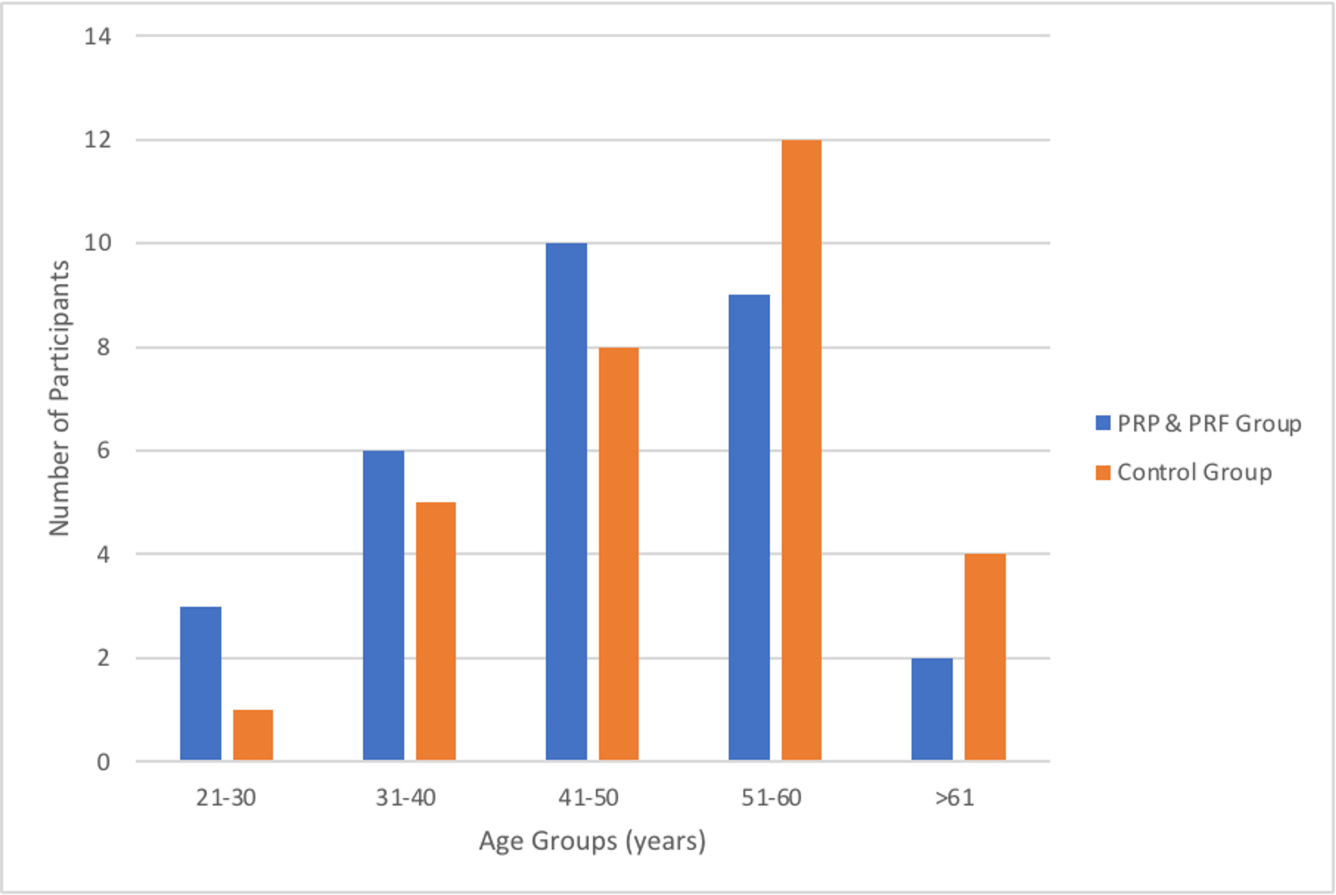
Age distribution in the PRP & PRF group and the conventional dressing group. PRP: platelet-rich plasma; PRF: platelet-rich fibrin.

**Figure 6:**
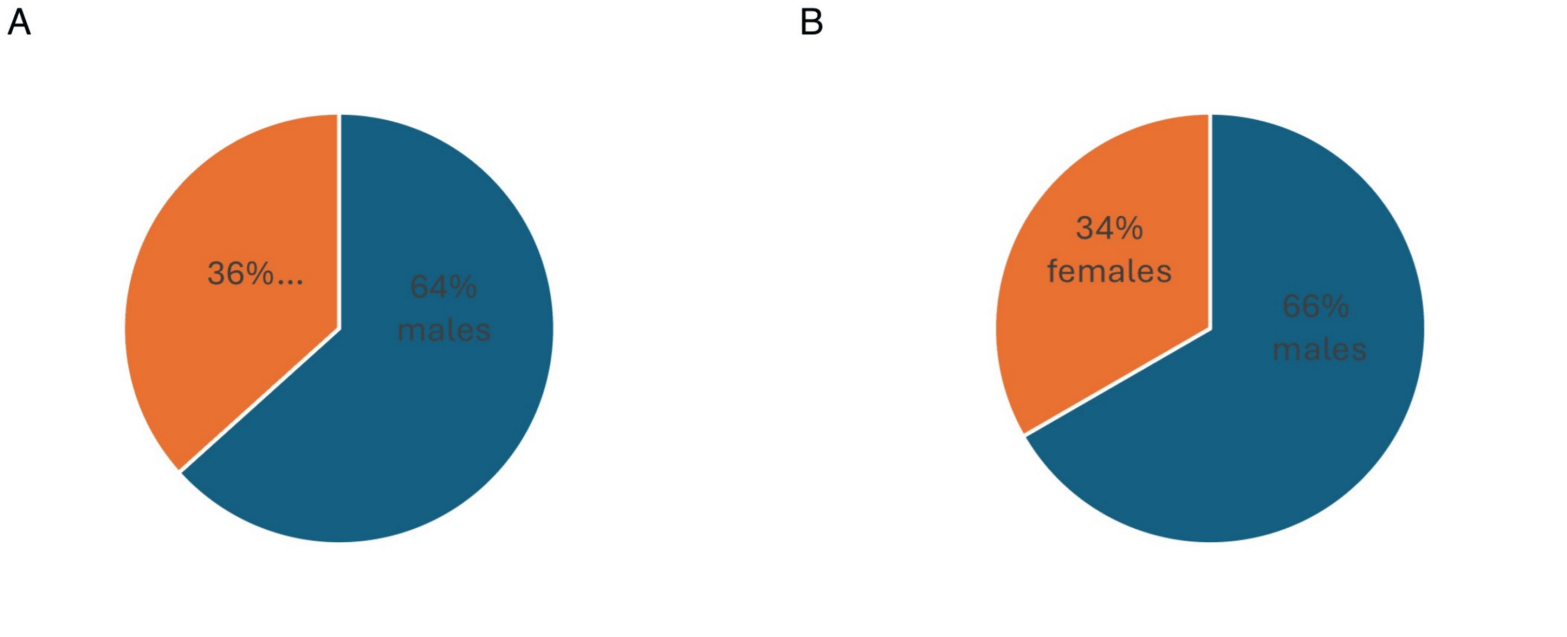
Pie chart representing gender distribution. Gender distribution in (A) the PRP & PRF group and (B) the conventional dressing group. PRP: platelet-rich plasma; PRF: platelet-rich fibrin.

The demographic analysis revealed that the mean age of the participants was 45.57 ± 9.33 years, with ages ranging from 25 to 65 years (Table 1 and Figure 5). The gender distribution showed a significant male predominance with 64% and 66% male patients compared to 36% and 34% in the PRP & PRF group and conventional group, respectively (Table 2 and Figure 6).

**Table 1:** Age distribution of the PRP & PRF group and the conventional dressing group. PRP: platelet-rich plasma; PRF: platelet-rich fibrin.

Age distribution	PRP & PRF group	Age distribution percentage in the PRP & PRF group	Conventional group	Age distribution percentage in the conventional group
21 – 30 years	3	10%	1	3.33%
31 – 40 years	6	20%	5	16.66%
41 – 50 years	10	33.33%	8	26.66%
51 – 60 years	9	30%	12	40%
>60 years	2	6.66%	4	13.33%
Total	30	100%	30	100%

**Table 2:** Gender distribution. PRP: platelet-rich plasma; PRF: platelet-rich fibrin.

Gender distribution	PRP & PRF group	Gender percentage in the PRP & PRF group	Conventional group	Gender percentage in the conventional group
Male	19	36%	20	34%
Female	11	64%	10	66%
Total	30	100%	30	100%

Clinical parameters

**Figure 7:**
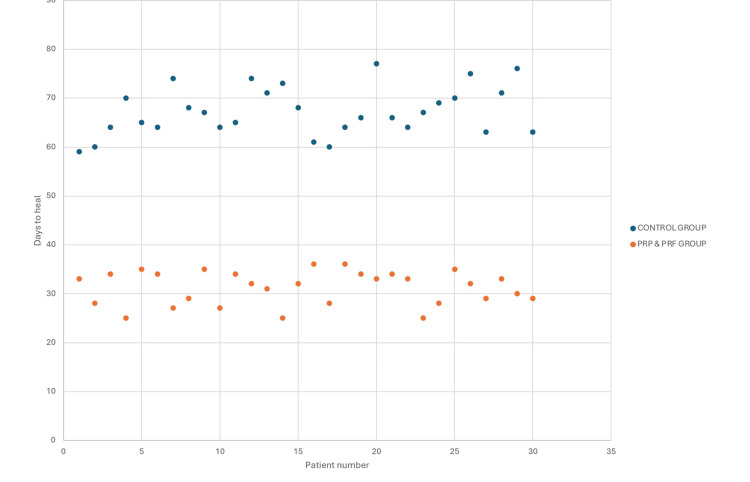
Comparison of duration of healing in days between the conventional and PRP & PRF groups. PRP: platelet-rich plasma; PRF: platelet-rich fibrin.

**Figure 8:**
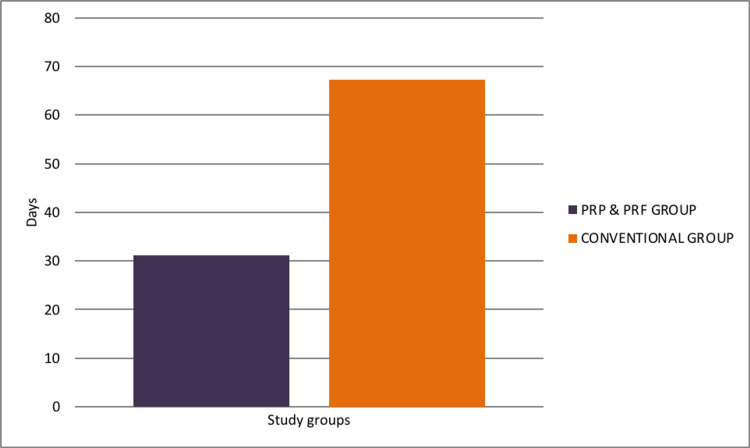
Bar graph representing the comparison between the mean duration of healing in the PRP & PRF group and the conventional group. PRP: platelet-rich plasma; PRF: platelet-rich fibrin.

In the present study involving 60 patients, 30 were treated with conventional cleaning and dressing methods, while 30 were treated with PRP & PRF dressings. The average healing duration in the study was significantly shorter for the PRP & PRF group. The mean healing time for this group was 4.45 weeks (31.2 ± 3.07 days) compared to 9.61 weeks (67.27 ± 9.19 days) for the conventional dressing group (Figures 7, 8). An unpaired t-test was applied to the two study groups and the difference in healing times was calculated, which was deemed to be statistically significant (p < 0.05), indicating that the PRP & PRF group promotes faster healing in comparison.

**Figure 9:**
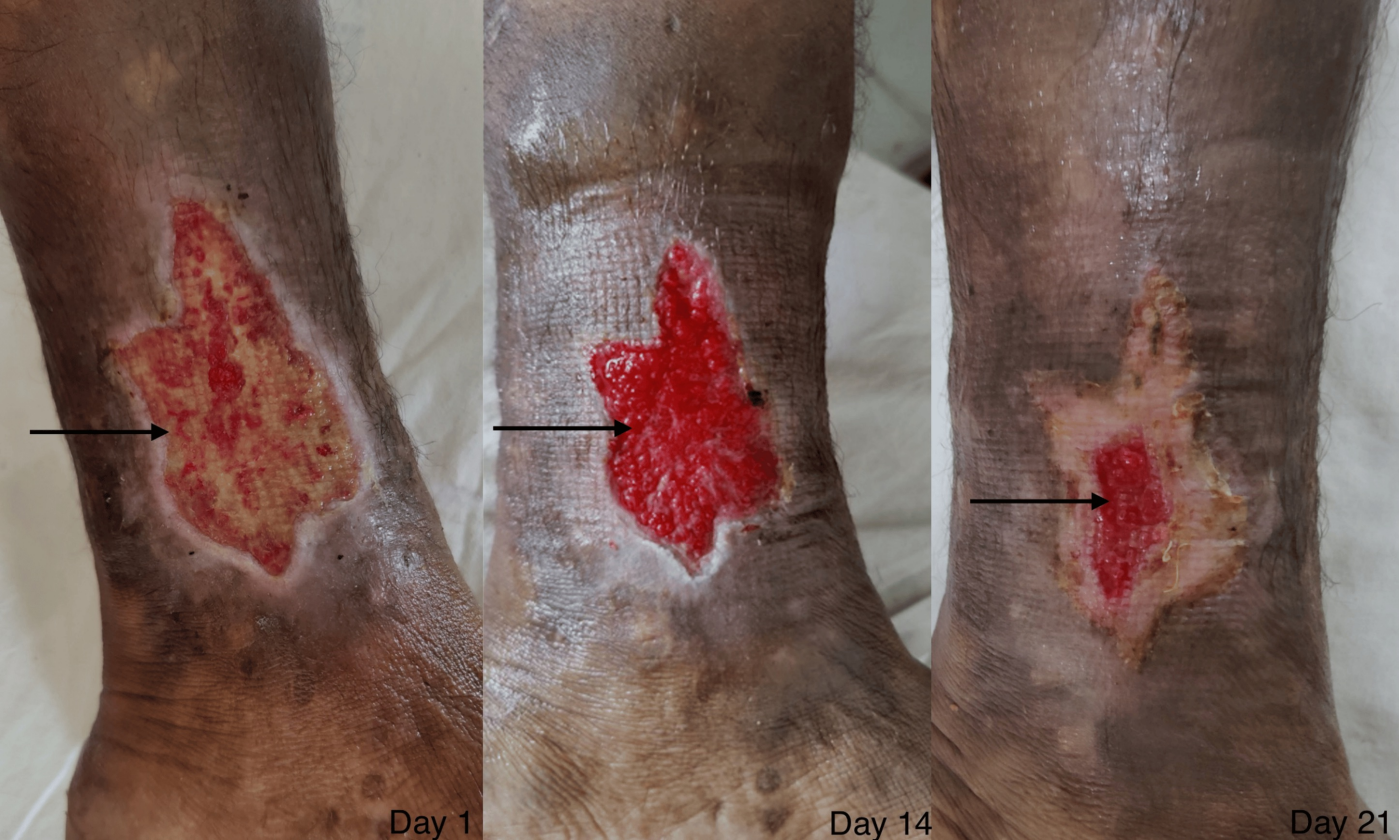
The wound of a patient on day one versus day 14 versus day 21 in the PRP & PRF group. The image shows the wound of a patient on day one, day 14, and day 21, depicting granulation tissue (black arrow) and reduction in size in the PRP & PRF group. PRP: platelet-rich plasma; PRF: platelet-rich fibrin.

**Figure 10:**
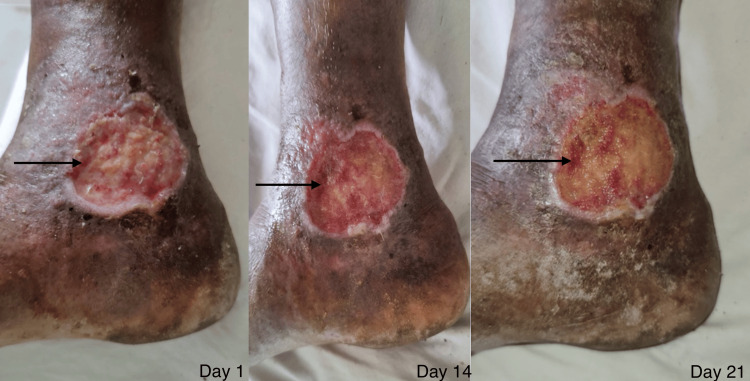
The wound of a patient on day one versus day 14 versus day 21 in the conventional dressing group. The image shows the wound of a patient on day one, day 14, and day 21, depicting granulation tissue (black arrow) and size reduction in the conventional dressing group.

**Figure 11:**
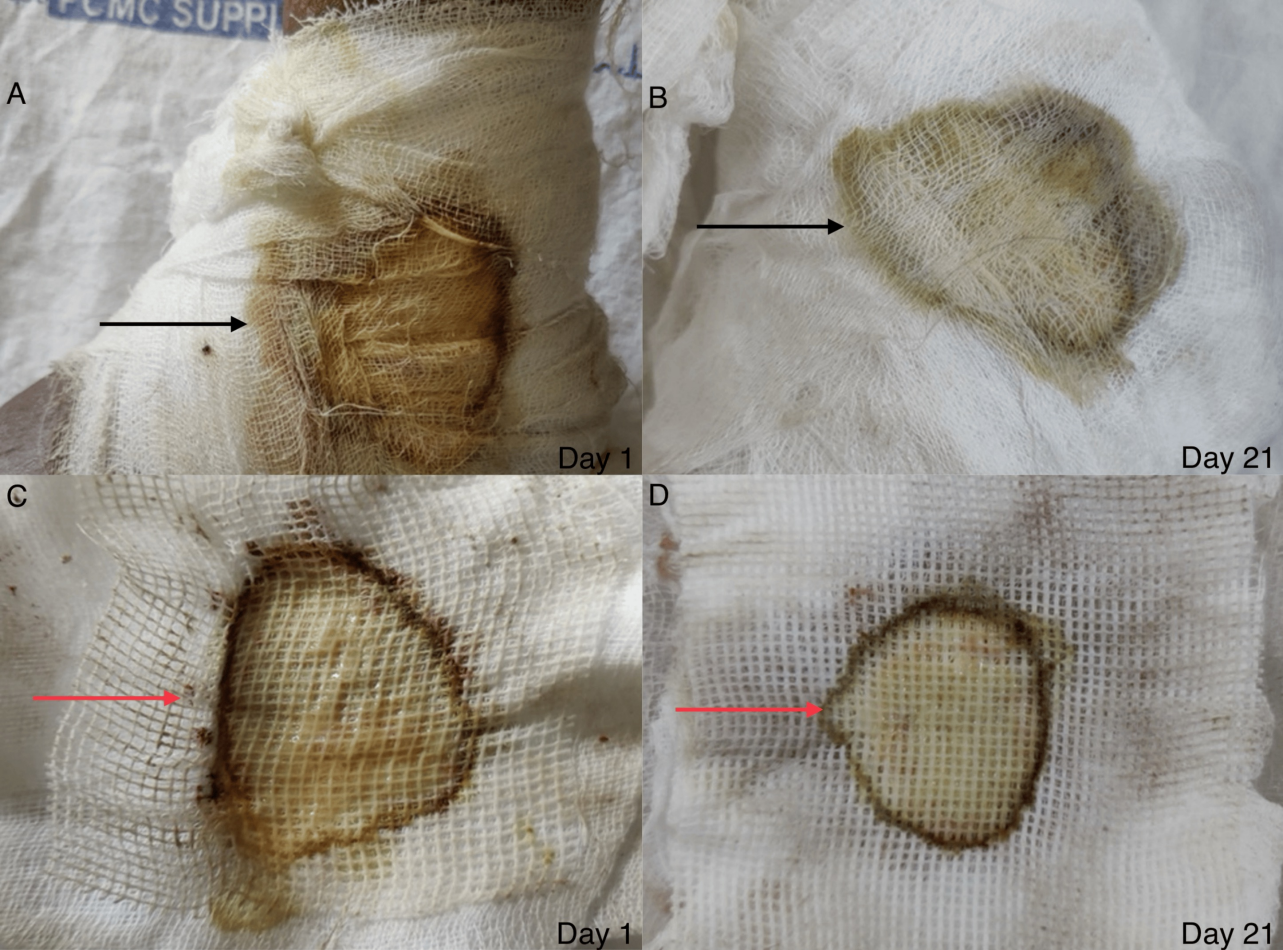
Comparison of dressing soakage of a patient on day one versus day 21. The image shows the comparison of dressing soakage from outside on (A) day one and (B) day 21 (black arrows), and from the inner side on (C) day one and (D) day 21 (red arrows).

For the healing rate, both case and control groups were compared at seven, 14, 21, and 28 days (Figures 9, 10). There was a reduction in soakage in both groups with serial dressings, but the case group had more reduction in soakage compared to the control group. Gauze pieces of 15 x 15 cm^2^ size, folded thrice from the middle, were used as a standard for dressing for every patient. On the 7th day, the case group had two gauze soakages compared to three in the control group, which subsequently reduced to half a gauze in the case group compared to one and a half gauze in the control group by the 21st day (Figure 11). However, the reduction in soakage could be attributed to a decrease in wound size, as there was a rapid decrease in wound size in the case group as compared to the control group.

The case group developed granulation faster as compared to the control group. The depth of the wound decreased faster in the case group and the color of the granulation tissue was bright red in the case group compared to the pale granulation tissue in the control group when compared on 21 days (Figures 9, 10). The wound edges and surrounding skin responded faster in the case group as compared to the control group. The signs of inflammation reduced faster in the case group compared to the control group.

**Figure 12:**
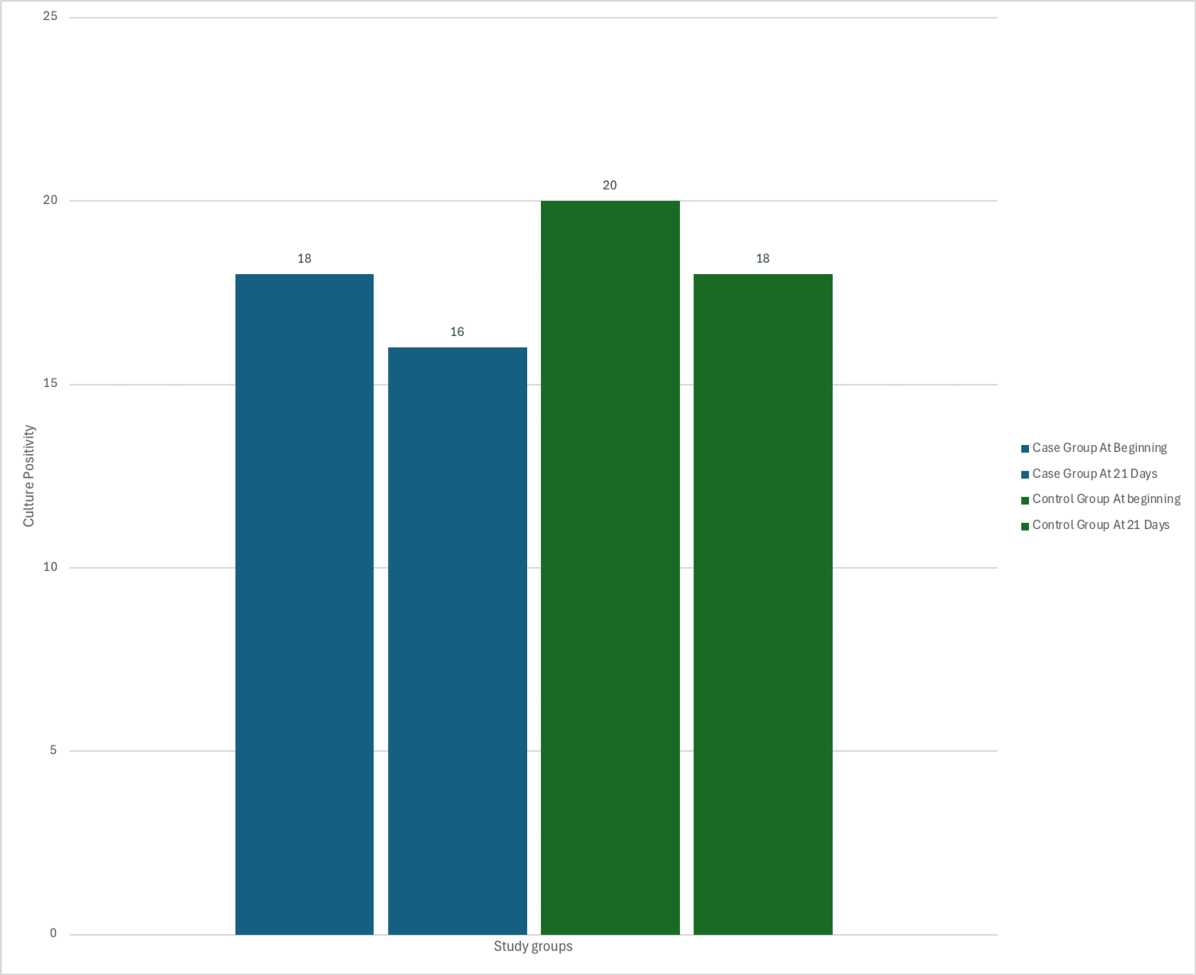
Bar graph showing comparison of culture positivity in the PRP & PRF group and the conventional group at the beginning of the study and at 21 days. PRP: platelet-rich plasma; PRF: platelet-rich fibrin.

Wound cultures were taken at the beginning of the study and were repeated on days 14 and 21 of the study. The table presents the significance of changes in culture positivity rates for the two groups over time. In the conventional dressing group, the proportion of culture-positive samples decreased from 0.667 at the beginning to 0.600 at the end. Similarly, in the PRP & PRF dressing group, the proportion decreased from 0.600 to 0.533. However, the differences in proportions (0.067 for both groups) were not statistically significant, as indicated by p-values greater than the conventional threshold of 0.05 (Table 3 and Figure 12).

**Table 3:** Comparison of culture positivity in the PRP & PRF group and the conventional dressing group. PRP: platelet-rich plasma; PRF: platelet-rich fibrin.

		Total samples	Culture positive	Proportion of culture positive to total samples	Difference between proportions	95% CI for difference	p-value
Conventional group	At beginning	30	20	0.667	0.067	(-0.177, 0.3099)	0.591
	At 21 days	30	18	0.600			
PRP & PRF group	At beginning	30	18	0.600	0.067	(-0.184, 0.317)	0.602
	At 21 days	30	16	0.533			

These results suggest that the observed changes in culture positivity rates over time were not statistically significant for either group. The infective foci present in the wound did not respond to the type of dressing but responded to antibiotics as per sensitivity.

## Discussion

Chronic wounds are defined by their inability to progress through the normal stages of healing, resulting in a protracted and pathological state of inflammation, which consequently leads to elevated morbidity and mortality rates [10,11]. The development of chronic wounds involves a multifaceted and intricate pathophysiological process, wherein underlying conditions such as diabetes mellitus, venous and arterial disease, and others contribute to the onset of ischemia and hypoxia through various mechanisms. This, in turn, triggers the activation of local reactive oxygen species and inflammatory cascades, which further impede the healing process. Additionally, the tissue damage inflicted by local inflammation is compounded by wound infections, leading to a profound disruption of the healing trajectory [12,13]. A self-perpetuating cycle ensues, wherein the recurrent stimulation of ischemic and necrotic tissue triggers a vicious loop of excessive inflammation, leading to further tissue deterioration and ultimately, the establishment of chronic wounds. This cycle of inflammation and tissue damage reinforces itself, hindering the wound's ability to progress through the normal healing stages.

Platelets exert a regulatory influence on inflammatory processes at the site of tissue damage through the controlled secretion of bioactive molecules, including growth factors (such as insulin-like growth factor, epidermal growth factor, and vascular endothelial growth factor) and chemokines, thereby modulating the inflammatory response and facilitating tissue repair. Therefore, in recent years, there has been an increased focus on the use of PRP and PRF as an advanced method to promote healing. Such platelet-rich preparations are a safe, easy, and cost-efficient method of accelerating healing and repair [14].

Although the use of platelet derivatives for the treatment of skin wounds has a five-decade history with various names, the first-known accepted description of the regenerative use of platelets was provided by Marx in 1998 as PRP [15].

PRP functions as a tissue sealant and drug delivery system [4], with the platelets initiating wound repair by releasing locally acting growth factors and cytokines [16]. A higher concentration of growth factors promotes the regeneration of epithelial and endothelial cells, stimulates angiogenesis, and collagen deposition, and accelerates the healing process [17].

Choukroun et al. [18] developed PRF in 2001 in France. PRF is a bioactive matrix comprising platelets, leukocytes, and a fibrin scaffold, which entraps and gradually releases cytokines, growth factors, and cellular constituents, thereby augmenting the wound-healing process. Furthermore, the autologous origin, ease of preparation, exemplary safety profile, and low cost of PRF render it a readily accessible and attractive therapeutic modality for clinical application.

This study’s clinical parameters indicated the mean duration of healing for the PRP & PRF group was 4.45 weeks (31.2 ± 3.07 days) compared to 9.61 weeks (67.27± 9.19 days) for the conventional dressing group. These findings are consistent with a recent meta-analysis of PRP in chronic refractory wounds by Shanqiang Qu et al. (2022), including 17 randomized controlled trials, which demonstrated that compared with the control group, PRP significantly increased the percentage of healed wounds and the percentage of the healed area [19].

The findings in this study with a mean healing duration of 4.45 weeks are better compared to seven weeks as reported by Pravin et al. (2016) and Somani et al. (2017). This can be attributed to the dual use of PRP & PRF in this study compared to the use of only PRF in the above-mentioned studies. Numerous clinical studies have consistently demonstrated the efficacy of PRF in the treatment of chronic wounds, particularly those of venous origin. Recent studies published in 2016, 2017, and 2018 have shown accelerated wound closure and healing outcomes with PRF application. Notably, PRF treatment has been reported to expedite the healing process by seven days compared to conventional therapy. Furthermore, studies have observed significant improvements in tissue restoration with PRF, with 73.3% of wounds showing restoration after PRF treatment compared to 53.3% with conventional treatment. Additionally, PRF application has been shown to achieve 85.51% wound restoration after four weeks and 100% restoration after seven weeks, surpassing conventional treatment outcomes of 42.74% and 42.6%, respectively [8,9].

A study conducted by Danielsen et al. (2008) found no statistically significant differences in the rate of epithelialization between acute surgical wounds treated conventionally (control group) and those treated with PRF. However, patients in the PRF group reported significantly reduced pain levels compared to the control group, suggesting a beneficial effect of PRF on postoperative pain management [20].

This study’s clinical parameters indicated that the PRP & PRF group did not have significant antibacterial action when compared to the conventional dressing group. These findings are consistent with the study of Shih-Chun Yang et al. (2023), who demonstrated that PRP might have some bacteriostatic activity against specific bacteria in varied dilutions but is unlikely to be a widespread approach against all species [21].

The limitations associated with this study are higher costs and difficulty in preparation of the PRP and PRF since centrifugation machines are not readily available at all levels of the medical healthcare system.

## Conclusions

PRP and PRF belong to a new generation of platelet concentrates that safely, cost-efficiently, and effectively enhance recovery. Activated platelets release multiple growth factors and cytokines that are involved in promoting tissue repair and regeneration.

They significantly reduce healing duration, improving the quality of life of patients, almost eliminating the morbidity of operative procedures, representing a breakthrough in the management of chronic wounds.

## References

[1] Brunicardi F, Andersen DK, Billiar TR (2019). Schwartz's Principles of Surgery, 11th edition. https://accessmedicine.mhmedical.com/book.aspx?bookID=2576.

[2] Marx RE (2004). Platelet-rich plasma: evidence to support its use. J Oral Maxillofac Surg.

[3] Chicharro-Alcántara D, Rubio-Zaragoza M, Damiá-Giménez E, Carrillo-Poveda JM, Cuervo-Serrato B, Peláez-Gorrea P, Sopena-Juncosa JJ (2018). Platelet rich plasma: new insights for cutaneous wound healing management. J Funct Biomater.

[4] Eppley BL, Woodell JE, Higgins J (2004). Platelet quantification and growth factor analysis from platelet-rich plasma: implications for wound healing. Plast Reconstr Surg.

[5] Ozer K, Colak O (2019). Leucocyte- and platelet-rich fibrin as a rescue therapy for small-to-medium-sized complex wounds of the lower extremities. Burns Trauma.

[6] Mosesson MW, Siebenlist KR, Meh DA (2001). The structure and biological features of fibrinogen and fibrin. Ann N Y Acad Sci.

[7] Dohan DM, Choukroun J, Diss A, Dohan SL, Dohan AJ, Mouhyi J, Gogly B (2006). Platelet-rich fibrin (PRF): a second-generation platelet concentrate. Part I: technological concepts and evolution. Oral Surg Oral Med Oral Pathol Oral Radiol Endod.

[8] Pravin AJS, Sridhar V, Srinivasan BN (2016). Autologous platelet rich plasma (PRP) versus leucocyte-platelet rich fibrin (L-PRF) in chronic non-healing leg ulcers--a randomised, open labelled, comparative study. J Evol Med Dent Sci.

[9] Somani A, Rai R (2017). Comparison of efficacy of autologous platelet-rich fibrin versus saline dressing in chronic venous leg ulcers: a randomised controlled trial. J Cutan Aesthet Surg.

[10] Olsson M, Järbrink K, Divakar U, Bajpai R, Upton Z, Schmidtchen A, Car J (2019). The humanistic and economic burden of chronic wounds: a systematic review. Wound Repair Regen.

[11] Martinengo L, Olsson M, Bajpai R (2019). Prevalence of chronic wounds in the general population: systematic review and meta-analysis of observational studies. Ann Epidemiol.

[12] Ammons MC, Morrissey K, Tripet BP (2015). Biochemical association of metabolic profile and microbiome in chronic pressure ulcer wounds. PLoS One.

[13] Canesso MC, Vieira AT, Castro TB (2014). Skin wound healing is accelerated and scarless in the absence of commensal microbiota. J Immunol.

[14] Xie J, Fang Y, Zhao Y, Cao D, Lv Y (2020). Autologous platelet-rich gel for the treatment of diabetic sinus tract wounds: a clinical study. J Surg Res.

[15] Marx RE, Carlson ER, Eichstaedt RM, Schimmele SR, Strauss JE, Georgeff KR (1998). Platelet-rich plasma: growth factor enhancement for bone grafts. Oral Surg Oral Med Oral Pathol Oral Radiol Endod.

[16] Everts PA, Brown Mahoney C, Hoffmann JJ, Schönberger JP, Box HA, van Zundert A, Knape JT (2006). Platelet-rich plasma preparation using three devices: implications for platelet activation and platelet growth factor release. Growth Factors.

[17] Jee CH, Eom NY, Jang HM (2016). Effect of autologous platelet-rich plasma application on cutaneous wound healing in dogs. J Vet Sci.

[18] Choukroun J, Adda F, Schoeffler C, Vervelle A (2001). Une opportunite en paro-implantologie: le PRF. (Article in French). Implantodontie.

[19] Qu S, Hu Z, Zhang Y (2022). Clinical studies on platelet-rich plasma therapy for chronic cutaneous ulcers: a systematic review and meta-analysis of randomized controlled trials. Adv Wound Care (New Rochelle).

[20] Danielsen P, Jørgensen B, Karlsmark T, Jorgensen LN, Ågren MS (2008). Effect of topical autologous platelet-rich fibrin versus no intervention on epithelialization of donor sites and meshed split-thickness skin autografts: a randomized clinical trial. Plast Reconstr Surg.

[21] Yang SC, Lin CF, Alshetaili A, Aljuffali IA, Chien MY, Fang JY (2023). Combining the dual antibacterial and regenerative activities of platelet-rich plasma with β-lactams to mitigate MRSA-infected skin wounds. Biomed Pharmacother.

